# Dataset of open-source software developers labeled by their experience level in the project and their associated software metrics

**DOI:** 10.1016/j.dib.2022.108842

**Published:** 2022-12-19

**Authors:** Quentin Perez, Christelle Urtado, Sylvain Vauttier

**Affiliations:** EuroMov Digital Health in Motion, Univ. Montpellier & IMT Mines Ales, Ales, France

**Keywords:** Empirical software engineering, GitHub contributors, Software metrics, Experienced developers, Software architecture, Java, Spring, Maven

## Abstract

Developers are extracted from 17 open-source projects from GitHub. Projects are chosen that use the java programming language, the Spring framework and Maven/Gradle build tools. Along with these developers, 24 software engineering metrics are extracted for each of them. These metrics are either calculated by analyzing the source code or relative to project management metadata. Each of these developers then are manually searched for in professional social media such as LinkedIn or Twitter to be labeled with their experience level in their project. Outliers are statistically detected and manually re-assigned when needed. The resulting dataset contains 703 anonymized developers qualified by their 24 project-related software engineering metrics and labeled for their experience. It is suitable for empirical software engineering studies that need to connect developers’ level of experience to tangible software engineering metrics.

**Specifications table**

**Table:** 

Subject	Software Engineering
Specific subject area	Labeled dataset of developers extracted from GitHub open-source projects associated to 24 software metrics.
Type of data	Table (csv)
How the data were acquired	Software developers are extracted from 17 open-source software projects hosted on GitHub. In order to do so, we reuse and adapt the PyDriller [1] tool. Using PyDriller, we compute 24 software metrics attached to each developer for a given project. Then, we search for the experience level of each developer in professional social networks and project documentation.
Data format	Raw
Description of data collection	The dataset is a collection of 703 anonymized developers extracted from 17 open-source GitHub projects. 24 software metrics are associated to each developer of a project that are calculated based on the developer’s contributions to the project and on project metadata. The dataset is labelled with the experience level of each developer amongst one of the following: Experienced Software Engineer, Software Engineer, Bot, Other, Unknown.
Data source location	• GitHub • LinkedIn • Twitter • Documentation of the software projects
Data accessibility	Repository name: Zenodo Data identification number: 10.5281/zenodo.7011334 Direct URL to data: https://zenodo.org/record/7011334[2]
Related research article	Q. Perez, C. Urtado, S. Vauttier. Mining Experienced Developers in Open-source Projects. 17th International Conference on Evaluation of Novel Approaches to Software Engineering (ENASE), Apr 2022, Online. pp.443-452. https://dx.doi.org/10.5220/0011071800003176[3]

## Value of the Data

• This dataset contains more than 700 developers extracted from 17 open-source projects hosted on GitHub associated with 24 software metrics that are computed for each developer. The value of this dataset comes both from the size of the dataset (24 metrics for 703 developers) but also from the different information attached to each developer (metrics and experience levels).
• Developers in the dataset are manually labelled with one of the following labels: Experienced Software Engineer, Software Engineer, Bot, Other, Unknown. Quality of the labelling is improved by a statistical analysis followed by a manual inspection of outliers and a re-labelling when needed.
• Gathering information about software developers and more particularly their experience level in open-source projects is a cumbersome task. Hence, this dataset might be of interest to researchers in software engineering.
• The dataset can be used to perform empirical studies in software engineering, more precisely about characteristics of software developers or relations between project code quality and developers. Moreover, it can be used in machine learning approaches (either unsupervised or supervised) thanks to both the labelling and the number of software metrics associated to each developer.

## 1 Objective

This dataset was created in a context related to empirical software engineering and machine learning. The data has been extracted from open-source GitHub projects. It is related to a research article [3]. This dataset is provided openly to researchers working in empirical software engineering and machine learning, to ease their data collection, developer-related software metrics calculus and data labelling. This kind of dataset is rare in this context as it requires both heavy calculus and tedious manual indexing. It is important for us to share it widely with the scientific community. Furthermore, this article is important for reproducibility purposes, as it clearly documents the retrieval process of the data used in the companion research article [3]. Also, the dataset could be used as a benchmark for comparing the performance of future research in this field. By officially publishing this dataset through Data In Brief, authors wish to advertise the solid conception of this dataset.

## 2 Data Description

The dataset of experienced developers is composed of 703 developers extracted from 17 open-source project hosted on GitHub [4]. Selected GitHub projects are mainly written in Java and all use the Java Spring Framework [5]. This framework provides languages (such as a deployment descriptor XML dialect and Java annotations) that support the definition of the architecture that will be automatically instantiated by the system to execute an application. Projects also use the Gradle [6] and Maven [7] automatic software management and automation tools. The use of these technologies is a deliberate choice in order to constitute a dataset of developers working with a Java ecosystem (Gradle/Maven, Java, *etc.*), Spring and GitHub. Table 1 provides metadata on the 17 selected projects: their total number of developers, their number of stars in GitHub, their GitHub creation date and their URL. The numbers of both developers and stars vary with time. Values in Table 1 are those retrieved on 2021/09/22. Criteria for selection are described below (in Section **Experimental design, materials and methods**).

**Table 1:** Metadata on projects in the dataset.

Project	#Developers	#Stars	Creation date	URL

Activiti	152	8012	2012-09	https://github.com/Activiti/Activiti
BroadleafCommerce	61	1490	2011-12	https://github.com/BroadleafCommerce/BroadleafCommerce
Camunda-bpm-spring-boot-starter	29	287	2013-01	https://github.com/camunda/camunda-bpm-spring-boot-starter
Dhis2-core	64	210	2016-08	https://github.com/dhis2/dhis2-core
Flowable-engine	199	4385	2016-10	https://github.com/flowable/flowable-engine
Jetcache	11	3035	2017-04	https://github.com/alibaba/jetcache
Moduliths	7	568	2018-05	https://github.com/odrotbohm/moduliths
Piggymetrics	13	10820	2015-03	https://github.com/sqshq/piggymetrics
Problem-spring-web	17	734	2015-08	https://github.com/zalando/problem-spring-web
Spring-boot-admin	94	10183	2014-07	https://github.com/codecentric/spring-boot-admin
Spring-petclinic	47	7454	2013-01	https://github.com/spring-projects/spring-petclinic
Spring-social	27	618	2011-02	https://github.com/spring-projects/spring-social
Spring-social-facebook	23	242	2011-05	https://github.com/spring-projects/spring-social-facebook
Spring-social-linkedin	7	71	2011-05	https://github.com/spring-projects/spring-social-linkedin
Springfox	128	5330	2012-05	https://github.com/springfox/springfox
UPortal	66	222	2011-10	https://github.com/uPortal-Project/uPortal
Ureport	6	1432	2017-06	https://github.com/youseries/ureport

Developers from those 17 projects are extracted using the GitHub API [8]. For each developer of each project, 24 metrics, described in Table 2, are computed.

**Table 2:** Description of the 24 computed metrics.

Kind of metric	Kind of element measured	Metric Code	Metric
Code metrics	Java Structure	AB	Number of ABstract classes created by a given developer
		NAB	Number of Non ABstract classes created by a given developer
		CII	Number of Classes Implementing an Interface created by a given developer
		CNII	Number of Classes Not Implementing an Interface created by a given developer
		CE	Number of Classes Extending another class created by a given developer
		CNE	Number of Classes Not Extending another class created by a given developer
		IEI	Number of Interfaces Extending another Interface created by a given developer
		INEI	Number of Interfaces Not Extending another Interface created by a given developer
	Gradle/ Maven Structure	AddLGM	Lines added in Gradle or Maven files by a given developer
		DelLGM	Lines deleted in Gradle or Maven files by a given developer in Gradle or Maven files
		ChurnLGM	Difference between added and deleted lines in Gradle / Maven files for a given developer
		NoMGM	Number of Modules Gradle or Maven created by a given developer
	Spring Architecture	AddSAM	Spring Architectural Modifications (lines specific to Spring) added by a given developer
		DelSAM	Spring Architectural Modifications (lines specific to Spring) by a given developer
		ChurnSAM	Difference between added and deleted specific Spring lines for a given developer
	Lines of Code	AddLOC	Number of Lines Of Code added by a given developer in project files
		DelLOC	Number of Lines Of Code deleted by a given developer in project files
		ChurnLOC	Difference between added and deleted lines of code in project files for a given developer
	Number of files	AddF	Number of Files added for a given developer
		DelF	Number of Files deleted for a given developer

Process Metrics		Followers	Numbers of GitHub followers of a given developer
		DiP	Days in Project. Number of days the developer has been in the project (time between first and last commit)
		IT	Inter-commit Time: Average time (in days) between two successive commits for a given developer
		NoC	Number of commit made by a developer

Four metrics (Number of Commits (NoC), Followers, Days in Project (DiP) and Inter-commit Time (ICT)) are process metrics (*i.e.* metrics monitoring the development process). The remaining 20 other metrics described in Table 2 are code metrics and are inherently related to source code. Code metrics measure different kinds of elements. Eight metrics are focused on the Java structure (*e.g.* Number of Abstract Classes (**AB**) or Number of Classes Implementing an Interface (**CII**)). Four metrics relate to the Gradle / Maven structure and three metrics measure the use of the Spring framework. Then, three metrics qualify the number of lines of code and two the number of files added or deleted.

**Fig. 1:**
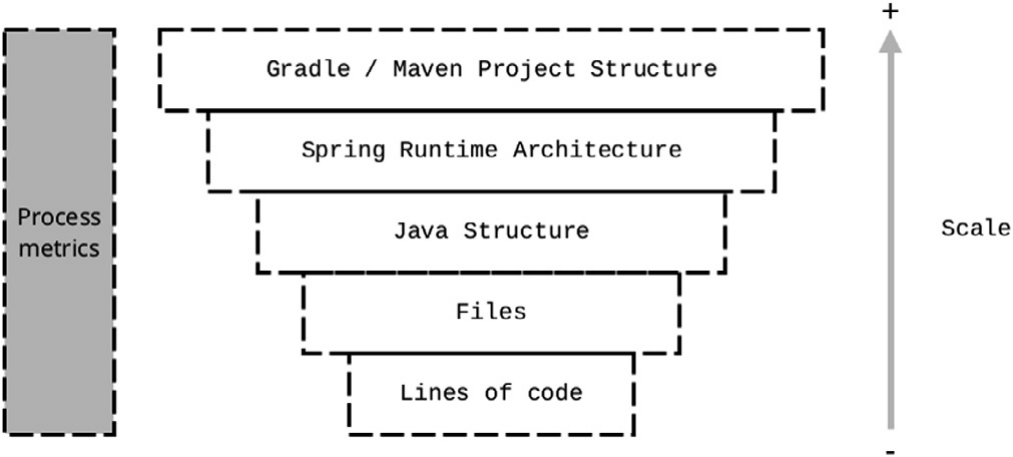
Software metrics hierarchy.

These metrics measure the software architecture at different scales (or granularities). Those scales are shown by Fig. 1. Moreover, to choose these metrics, we rely on the work of Di Bella et al. [9] and Perez et al. [10]. Di Bella et al. use an unsupervised method to classify developers in 4 groups from rare to core developers. They show that several metrics are discriminant for this classification: Number of Commits, Lines of Codes, Days in Project and Inter-commit Time. Hence, we choose to reuse these metrics to constitute our dataset. Perez et al. use Spring markers (specific Java annotations) to statistically distinguish categories of developers having an experience in working on the *runtime*architecture of the software. Therefore, we also choose to reuse their identified three variables specific to Spring *runtime*software architecture.

Table 3 statically describes the 24 metrics with figures computed on the whole dataset. For each metric, we compute its:

• Mean,
• Standard deviation (Std),
• Minimum (Min) and Maximum (Max) values,
• 1st (25%) and 3rd (75%) percentiles,
• and, Median.

**Table 3:** Statistical description for the 24 computed metrics.

	Variable											
	Followers	NoC	DiP	ICT	AB	NAB	CII	CNII	CE	CNE	INEI	IEI
Mean	187.96	90.89	428.60	30.00	7.12	121.05	45.07	83.10	58.51	69.66	9.34	7.57
Std	1325.23	362.28	948.31	79.70	5.34	953.37	358.38	651.86	505.44	508.25	68.85	69.27
Min	0.00	1.00	0.00	0.00	0.00	0.00	0.00	0.00	0.00	0.00	0.00	0.00
25%	2.00	1.00	0.00	0.00	0.00	0.00	0.00	0.00	0.00	0.00	0.00	0.00
Median	8.00	3.00	2.82	0.65	0.00	0.00	0.00	0.00	0.00	0.00	0.00	0.00
75%	34.00	13.00	381.45	16.92	0.00	3.00	0.00	2.00	0.50	2.00	0.00	0.00
Max	21837.00	4094.00	6774.00	836.49	1088.00	19449.00	7044.00	13493.00	10771.00	9766.00	1255.00	1236

	Variable											
	AddLGM	DelLGM	ChurnLGM	NoMGM	AddLOC	DelLOC	ChurnLOC					

Mean	49.25	34.34	14.91	1.32	7836.38	1491.30	6345.07					
Std	249.59	168.42	176.03	10.28	61262.35	14009.69	51324.20					
Min	0.00	0.00	-1617.00	0.00	0.00	0.00	-1576.00					
25%	0.00	0.00	0.00	0.00	5.00	1.00	0.00					
Median	0.00	0.00	0.00	0.00	43.00	5.00	20.00					
75%	3.50	1.00	0.00	0.00	372.00	76.00	269.00					
Max	3948	2364.00	3126	186.00	1328791.00	228558.00	1100233.00					

	Variable											
	AddF	DelF	AddSAM	DelSAM	ChurnSAM							

Mean	72.60	53.78	39.04	27.82	11.23							
Std	540.05	590.00	468.42	352.76	157.73							
Min	0.00	0.00	0.00	0.00	-1473.00							
25%	0.00	0.00	0.00	0.00	0.00							
Median	0.00	0.00	0.00	0.00	0.00							
75%	3.00	0.00	0.00	0.00	0.00							
Max	11153.00	12663.00	11898.00	8630.00	3268.00							

We check that computed metrics are consistent, for instance that AB+NAB=CE+NCE. As seen in Table 3, metrics obey a large statistical dispersion due to some developers having a high level of seniority and therefore a high level of contribution in projects.

Developers in the dataset are manually labelled according to their experience level in their project, using one of the following labels:

• Experienced Software Engineer (ESE),
• Software Architect (SA),
• Software Engineer (SE),
• Non Software Engineer (NSE),
• Bot (BOT),
• Unknown (UNK).

**Fig. 2:**
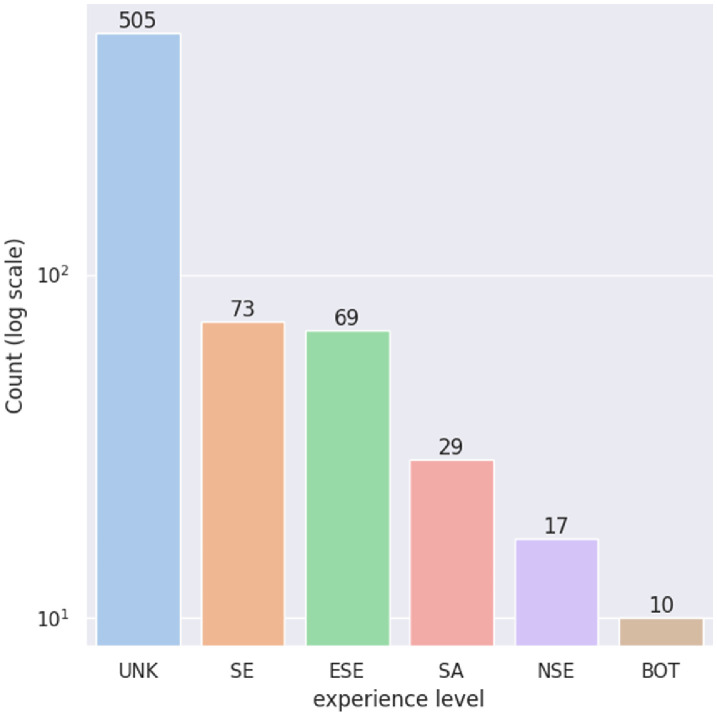
Number of developers per experience level in the dataset.

Labels are described below (in Section Experimental design, materials and methods). Fig. 2 presents the total number of developers per experience level. The major part (505 out 703 developers) of the dataset is composed of developers whose role was not found. This comes from the nature of the open-source projects where a large proportion of developers are very occasional or even contributed only once. In the other categories, except for the BOT category, there is a total of 188 developers whose experience level has been clearly identified. There is a good balance between software engineers (73) and experienced software engineers (69). 29 developers are software architects whereas 17 clearly identify as having a specific IT role (such as UX/UI designer or project manager) while not being developers. Finally, 10 developers are identified as BOTs, *i.e.* continuous integration systems such as Jenkins or Travis which automatically commit on GitHub repositories.

**Fig. 3:**
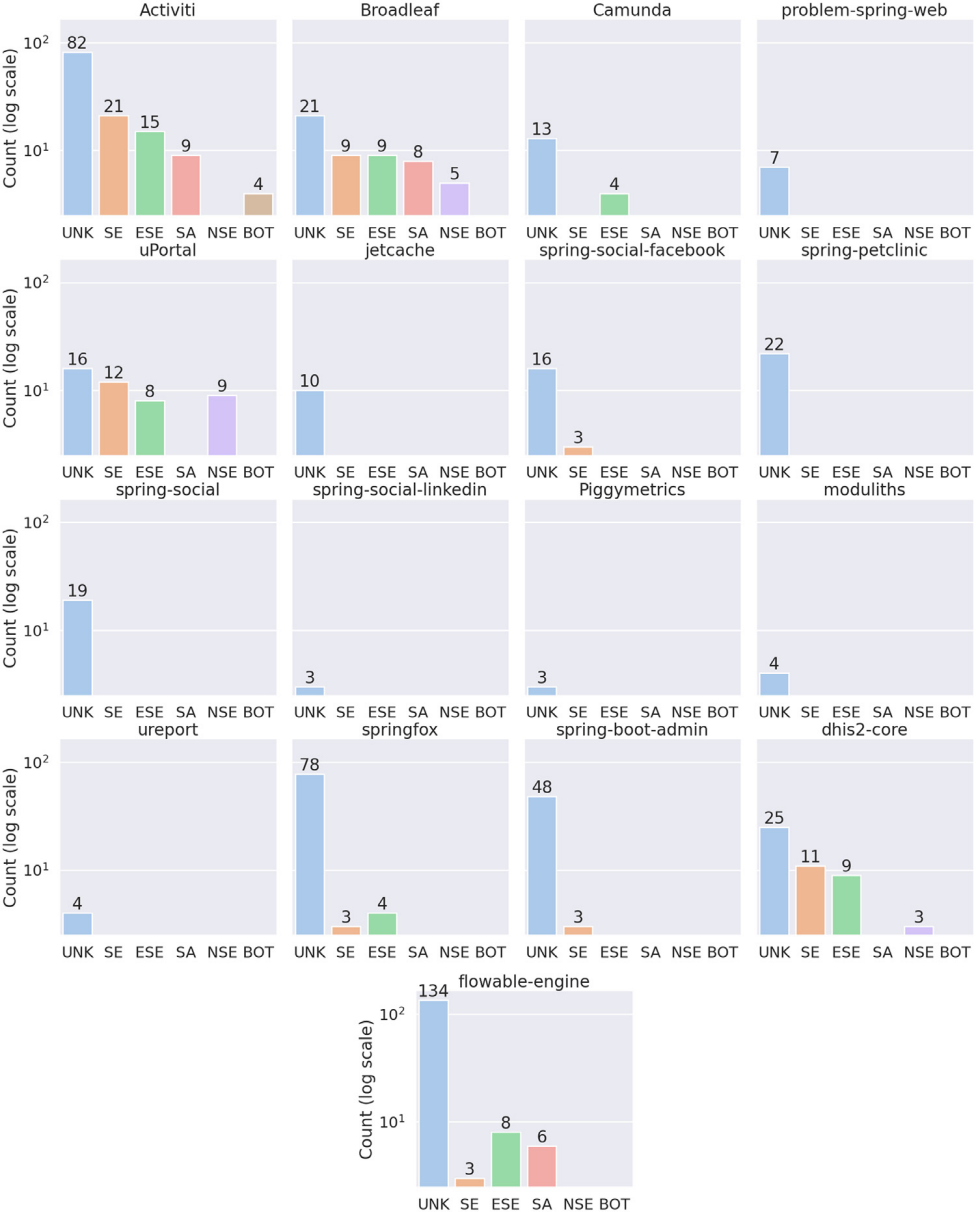
Number of developers per category for each project (represented using a logarithmic scale).

Fig. 3 shows the number of developers per experience level for each project (represented using a logarithmic scale). As described in Fig. 2, in all projects, a majority of developers have an unknown role (UNK). Four projects (Activiti, Broadleaf, dhis2-core and flowable-engine) have a plurality of developers (SE, ESE, SA, NSE, BOT). Others projects have only a few SE, ESE or SA.

## 3 Experimental Design, Materials and Methods

The data acquisition process, described using the Business Process Modeling and Notation (BPMN) [11], is shown in Fig. 5. The different steps of this data acquisition process are the following:

1. **GitHub project selection**: we manually select 17 projects from GitHub using the quality criteria given by Kalliamvakou et al. [12] for open source repository mining. We also add extra selection criteria to target projects that use the Spring Framework and have at least two developers.
2. Data acquisition process for developers (parallel tasks):
  (a) **Developers extraction from projects**: we extract the set of 951 developers from the 17 selected projects using the GitHub API. Each extracted developer is linked to its project. Thus, a developer appearing in two projects is considered different in each.
  (b) **Developers metadata retrieval**: extracted data about developers contain username, name and email as described in developers’ GitHub accounts.
3. Data acquisition process for metrics (parallel tasks):
  (a) **Source code retrieval**: for each project, we collect the source code.
  (b) **Commits retrieval**: we acquire project histories composed of the set of all commits from the first (date of the project creation on GitHub) to the latest (date of the dataset retrieval as given by the commit ID in Table 4).

**Table 4:** Latest selected commit for each project.

Project	Extraction commit ID	Commit date	Date of manual developer’s annotation
Activiti	77c0f3f27e293841398ae85465f613fe2b59afe	17-06-2021	21-03-2021
BroadleafCommerce	3628211ba9f36700e581a8b5e32a8c5423b5526	29-09-2020	24-03-2021
Camunda-bpm-spring-boot-starter	6df7d44acde821251109dc0d572dca6bb0b19d6e	23-10-2020	24-03-2021
Dhis2-Core	864a6db37966148cc5d72ba040e8843e84c90062	22-12-2020	24-03-2021
Flowable-Engine	e84c5889e078cb8aef83a9ffc7e545773d87d7b7	06-01-2021	26-03-2021
Jetcache	c549655f4fbf17eadf42c3a4bd266dee79fad8bc	13-10-2021	26-03-2021
Moduliths	df6bc564a117da97734b8eb016ade4ea2f8e94bb	03-11-2020	26-03-2021
Piggymetrics	fd5ee3c555ea9cd6067eacf3f2a3e8b85fe4fe77	19-01-2021	26-03-2021
Problem-spring-web	6f0c9bbb7d7e6f9a7af5b1c7c92cdd9d3cd3edeb	02-11-2020	26-03-2021
Spring-boot-admin	fb0041739c15975a42de508a202dbbe27f75cc27	11-11-2020	26-03-2021
Spring-petclinic	8b1ac6736e3347f34d79620170983fc4c99746cb	06-11-2020	26-03-2021
Spring-social	e41cfecb288022b83c79413b58f52511c3c9d4fc	04-04-2019	27-03-2021
Spring-social-facebook	ae2234d94367eaa3adbba251ec7790d5ba7ffa41	04-04-2019	27-03-2021
Spring-social-linkedin	0c181af6e5751a7588989415909d0ffaf1b79946	04-04-2019	27-03-2021
Springfox	ab5868471cdbaf54dac01af12933fe0437cf2b01	14-10-2020	27-03-2021
UPortal	98e85d42c09f7e7d2113b062a9cda82d431fbe48	02-11-2020	27-03-2021
Ureport	07f9c32593274c1f23e403ffddcb86ffb9964799	26-09-2020	27-03-2021
4. **Metrics computation for each developers**: using a modified version of the PyDriller tool [1], we compute 24 metrics described in Table 2. For each project and developer, metrics are computed using the whole project history extracted at Step 2. Table 5 presents 4 global metrics characterizing the extracted software projects. We use the cloc software [13] to compute the number of files and lines of code listed in Table 5. Table 4 also gives the number of developers present in the dataset for each project.

**Table 5:** Computed global metrics for each extracted project.

Project	Commits	Developers	LOC	Files
Activiti	10680	131	267281	4458
BroadleafCommerce	16706	52	373815	3468
Camunda-bpm-spring-boot-starter	641	22	11375	275
Dhis2-Core	7331	51	620885	6511
Flowable-Engine	12159	151	1580445	15212
Jetcache	932	11	17371	294
Moduliths	165	5	6563	147
Piggymetrics	159	4	19954	159
Problem-spring-web	1011	12	7794	204
Spring-boot-admin	1436	53	71526	787
Spring-petclinic	720	24	12889	81
Spring-social	1737	23	15677	292
Spring-social-facebook	1301	21	18536	420
Spring-social-linkedin	805	6	14515	261
Springfox	3755	85	118140	1406
UPortal	15794	47	215589	2541
Ureport	440	5	72301	731
5. **Data cleaning**: we perform a manual cleaning step to exclude developers that did not change at least one line, as synthesized in the following variables: AddLGM, DelLGM, AddLoC, DelLoC, AddSAM, DelSAM. When the sum of these six variables is equal to zero the developer is removed from the dataset. By this means, the dataset is reduced from 951 to 703 developers.
6. **Developer labelling**: each developer extracted from GitHub is mapped to its experience level in the project in a three stepped process (see Fig. 4). The labelling process mainly relies on a manual search on internet for each developer, using his / her GitHub username and name. We trust this labelling method because many developers use social networks [14]. We collect developers’ experience levels from LinkedIn [15], Twitter [16] and GitHub profiles or project documentation websites. When a developer’s GitHub name is found in one of those search engines, we check that the developer mentions that he / she is working on the given project (so as to prevent confusion with potential homonyms). The developer’s profile is manually read through to determine the developer’s label. The list of labels used to qualify developers’ experience is inspired from the 2021 Stack Overflow Developer Survey [17]. After this first step, a statistical analysis (isolation forest) is performed to detect labelling outliers with respect to their metrics values. Outliers are then reviewed manually again in a third step in order to check their labelling and correct it if needed.

**Fig. 4:**
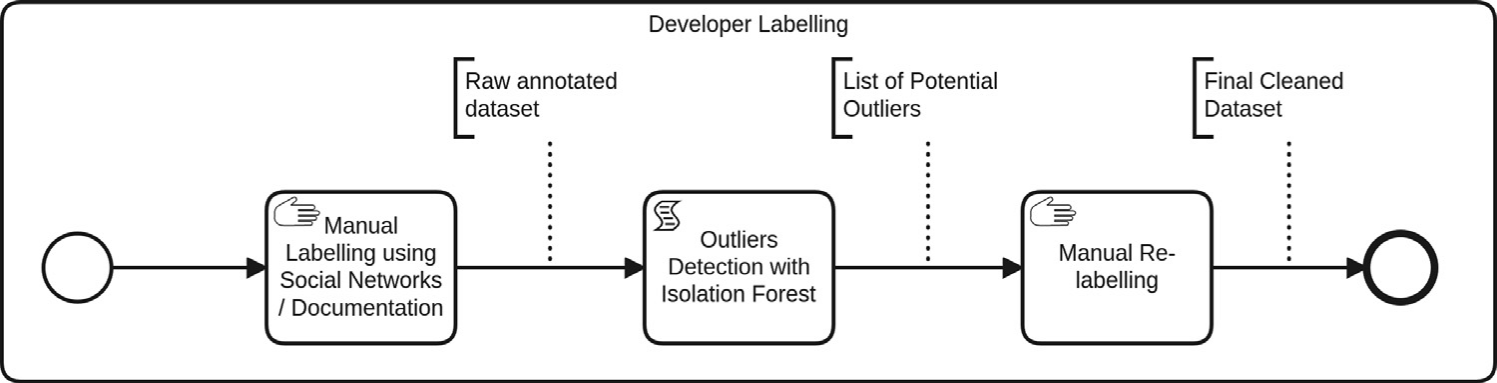
Labelling process modeled with BPMN.

**Fig. 5:**
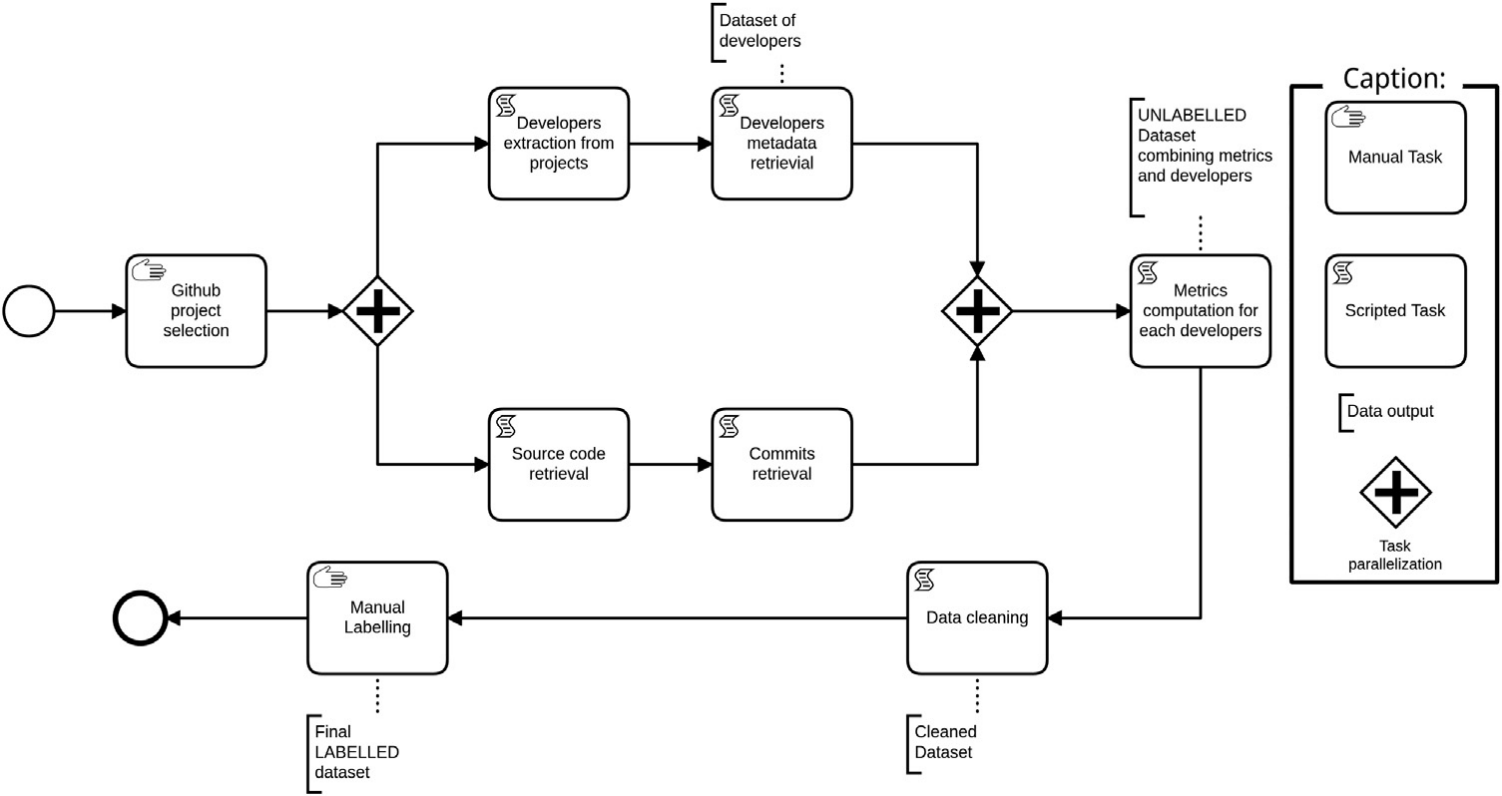
Data acquisition process modeled with BPMN.
  Following is a detailed description of this three step process:
  **Step 1: Manual labelling.**Each developer is searched for in LinkedIn, Twitter and GitHub profiles or project documentation websites using his / her GitHub username and name. When a developer is found in one of those search engines, we check that the developer mentions that he / she is working on the given project (so as to prevent confusion with potential homonyms). If the profile of a given developer mentions [17]:
  • “Architect” or “Senior Software Engineer” then the developer is labelled as ”Experienced Software Engineer” (ESE) [18],
  • “Junior Software Engineer” or “Software Engineer” then the developer is labelled as “Software Engineer” (SE),
  • “Developer” then we search if the developer has a Master of Sciences in Software Engineering. If so, the developer is labelled as “SE”; else the developer is labelled as “OTHER”.
  • Other descriptions than “SE” or “ESE” the developer is labelled as “OTHER”.
  Table 6 summarizes the keywords searched for in developers’ profiles to label them.

**Table 6:** Keywords and information used to label developers.

Keywords	Developer label
∘ “Architect” ∘ “Senior Architect”	Software Architect (SA)
∘ “Senior Software Engineer”	Experienced Software Engineer (ESE)
∘ “Junior Software Engineer” ∘ “Software Engineer”	Software Engineer (SE)
∘ “Developer” AND “MSc in Software Engineering”	Software Engineer (SE)
∘ “Developer”	Non Software Engineer (NSE)
∘ “Bot” (in GitHub username)	BOT
Other experience level	Non Software Engineer (NSE)
No information found	Unkwnon (UNK)
  **Step 2: Outliers detection.** To avoid misclassifications, we have sought outliers using an Isolation-Forest method. Indeed, we assume that equally labeled developers should have comparable metrics values, and conversely that developers from two different metrics profiles should be labelled differently. Isolation-Forest calculates a score for each observation in the dataset. This score provides a measure of normality for each observation and thus provide a set of possibly mislabeled developers.
  **Step 3: Manual relabelling.** After an inspection of potential outliers, we have manually relabeled 21 of them. This manual relabelling process increases the quality of the labelling.
  It is important to note that the dataset is enriched by manual labelling which makes it ready for supervised machine learning algorithms. However, users of the dataset might want to dismiss this labelling for unsupervised learning or might want to do a labelling of their own. In the latter cases, the dataset can still be considered a relevant contribution as it is rich of 24 calculated metrics.

## Ethics Statements

By its nature, the extracted data contains GitHub usernames associated to metrics and experience level in 17 projects. Code extraction and information relative to developers for each project on GitHub comply with the GitHub policies. Information gathered using social networks (Twitter, GitHub and LinkedIn) about developers are compliant with the platforms’ data distribution policies. Developers’ experience level provides information about developers’ skills. Hence, we fully anonymized the GitHub usernames. By doing so, it is very difficult to trace back to the non-anonymized developer by simple metric calculation. This computational difficulty combined with the fully anonymization of GitHub usernames guarantee developers’ anonymity.^1^

## CRediT authorship contribution statement

**Quentin Perez:** Conceptualization, Methodology, Software, Data curation, Writing – original draft. **Christelle Urtado:** Methodology, Writing – original draft, Supervision, Validation. **Sylvain Vauttier:** Methodology, Writing – original draft, Supervision, Validation.

## Declaration of Competing Interest

The authors declare that they have no known competing financial interests or personal relationships that could have appeared to influence the work reported in this paper.

## Data Availability

Dataset of Open-Source Software Developers Labeled by their Experience Level in the Project and their Associated Software Metrics (Original Data) (Zenodo).

## Footnotes

1 http://www.imt.fr
